# Assessing the Relationship of Ancient and Modern Populations

**DOI:** 10.1534/genetics.117.300448

**Published:** 2017-11-22

**Authors:** Joshua G. Schraiber

**Affiliations:** Department of Biology, and Institute for Genomics and Evolutionary Medicine, Temple University, Philadelphia, Pennsylvania 19122

**Keywords:** population continuity, diffusion theory, ancient DNA, SNP data

## Abstract

Genetic material sequenced from ancient samples is revolutionizing our understanding of the recent evolutionary past. However, ancient DNA is often degraded, resulting in low coverage, error-prone sequencing. Several solutions exist to this problem, ranging from simple approach, such as selecting a read at random for each site, to more complicated approaches involving genotype likelihoods. In this work, we present a novel method for assessing the relationship of an ancient sample with a modern population, while accounting for sequencing error and postmortem damage by analyzing raw reads from multiple ancient individuals simultaneously. We show that, when analyzing SNP data, it is better to sequence more ancient samples to low coverage: two samples sequenced to 0.5× coverage provide better resolution than a single sample sequenced to 2× coverage. We also examined the power to detect whether an ancient sample is directly ancestral to a modern population, finding that, with even a few high coverage individuals, even ancient samples that are very slightly diverged from the modern population can be detected with ease. When we applied our approach to European samples, we found that no ancient samples represent direct ancestors of modern Europeans. We also found that, as shown previously, the most ancient Europeans appear to have had the smallest effective population sizes, indicating a role for agriculture in modern population growth.

## Abstract (precis)

Sequencing DNA from deceased individuals can inform whether the individuals that currently live in a location are descended from individuals that.....

ANCIENT DNA (aDNA) is now ubiquitous in population genetics. Advances in DNA isolation (Dabney *et al.* 2013), library preparation (Meyer *et al.* 2012), bone sampling (Pinhasi *et al.* 2015), and sequence capture (Haak *et al.* 2015) make it possible to obtain genome-wide data from hundreds of samples (Allentoft *et al.* 2015; Haak *et al.* 2015; Mathieson *et al.* 2015; Fu *et al.* 2016). Analysis of these data can provide new insight into recent evolutionary processes, which leave faint signatures in modern genomes, including natural selection (Jewett *et al.* 2016; Schraiber *et al.* 2016) and population replacement (Lazaridis *et al.* 2014; Sjödin *et al.* 2014).

One of the most powerful uses of aDNA is to assess the continuity of ancient and modern populations. In many cases, it is unclear whether populations that occupied an area in the past are the direct ancestors of the current inhabitants of that area. However, this can be next to impossible to assess using only modern genomes. Questions of population continuity and replacement have particular relevance for the spread of cultures and technology in humans (Lazaridis *et al.* 2016). For instance, recent work showed that modern South Americans are descended from people associated with the Clovis culture that inhabited North America over 10,000 years ago, further enhancing our understanding of the peopling of the Americas (Rasmussen *et al.* 2014).

Despite its utility in addressing difficult-to-answer questions in evolutionary biology, aDNA also has several limitations. Most strikingly, DNA decays rapidly following the death of an organism, resulting in highly fragmented, degraded starting material when sequencing (Sawyer *et al.* 2012). Thus, ancient data are frequently sequenced to low coverage, and has a significantly higher rate of misleadingly called nucleotides than modern samples. When working with diploid data, as in aDNA extracted from plants and animals, the low coverage prevents genotypes from being called with confidence.

Several strategies are commonly used to address the low-coverage data. One of the most common approaches is to sample a random read from each covered site, and use that as a haploid genotype call (Skoglund *et al.* 2012; Allentoft *et al.* 2015; Haak *et al.* 2015; Mathieson *et al.* 2015; Fu *et al.* 2016; Lazaridis *et al.* 2016). Many common approaches to the analyses of aDNA, such as the usage of F-statistics (Green *et al.* 2010; Patterson *et al.* 2012), are designed with this kind of dataset in mind. F-statistics can be interpreted as linear combinations of simpler summary statistics, and can often be understood in terms of testing a tree-like structure relating populations. Nonetheless, despite the simplicity and appeal of this approach, it has several drawbacks. Primarily, it throws away reads from sites that are covered more than once, resulting in a potential loss of information from expensive, difficult-to-acquire data. Moreover, as shown by Peter (2016), F-statistics are fundamentally based on heterozygosity, which is determined by samples of size 2, and thus limited in power. Finally, these approaches are also strongly impacted by sequencing error, postmortem damage (PMD), and contamination.

On the other hand, several approaches exist to either work with genotype likelihoods or the raw read data. Genotype likelihoods are the probabilities of the read data at a site, given each of the three possible diploid genotypes at that site. They can be used in calculation of population genetic statistics, or likelihood functions, to average over uncertainty in the genotype (Korneliussen *et al.* 2014). However, many such approaches assume that genotype likelihoods are fixed by the SNP calling algorithm [although they may be recalibrated to account for aDNA-specific errors, as in Jónsson *et al.* (2013)]. However, with low coverage data, an increase in accuracy is expected if genotype likelihoods are coestimated with other parameters of interest, due to the covariation between processes that influence read quality and genetic diversity, such as contamination.

A recent method that coestimates demographic parameters, along with error and contamination rates, by using genotype likelihoods, showed that there can be significant power to assess the relationship of a single ancient sample to a modern population (Racimo *et al.* 2016). Nonetheless, they found that, for very low coverage data, inferences were not reliable. Thus, they were unable to apply their method to the large number of extremely low coverage (<1×) genomes that are available. Moreover, they were unable to explore the tradeoffs that come with a limited budget: can we learn more by sequencing fewer individuals to high coverage, or more individuals at lower coverage?

Here, we develop a novel maximum likelihood approach for analyzing low coverage aDNA in relation to a modern population. We work directly with raw read data and explicitly model errors due to sequencing and portmortem damage. Crucially, our approach incorporates data from multiple individuals that belong to the same ancient population, which we show substantially increases power and reduces error in parameter estimates. We then apply our new methodology to ancient human data, and show that we can perform accurate demographic inference, even from very low coverage samples, by analyzing them jointly.

## Methods

### Sampling alleles in ancient populations

We assume a scenario in which allele frequencies are known with high accuracy in a modern population. Suppose that an allele is known to be at frequency x∈(0,1) in the modern population, and we wish to compute the probability of obtaining *k* copies of that allele in a sample of *n* (0≤k≤n) chromosomes from an ancient population. As we show in the *Appendix*, conditioning on the frequency of the allele in the modern population minimizes the impact of ascertainment, and allows this approach to be used for SNP capture data.

To calculate the sampling probability, we assume a simple demographic model, in which the ancient individual belongs to a population that split off from the modern population $\tau_{1}$ generations ago, and subsequently existed as an isolated population for $\tau_{2}$ generations. Further, we assume that the modern population has effective size $N_{e}^{\left(1\right)},$ and that the ancient population has effective size $N_{e}^{\left(2\right)},$ and measure time in diffusion units, $t_{i}=\tau_{i}/\left(2N_{e}^{\left(i\right)}\right).$ If we know the conditional probability that an allele is at frequency *y* in the ancient sample, given that it is at frequency *x* in the modern population, denoted $f\left(y;x,t_{1},t_{2}\right),$ then the sampling probability is simply an integral,

$$\begin{array}{c} P_{n,k}\left(x\right)=\int_{0}^{1}\left(\begin{array}{c} n\\ k \end{array}\right)y^{k}{\left(1-y\right)}^{n-k}f\left(y;x,t_{1},t_{2}\right)dy\\ =\left(\begin{array}{c} n\\ k \end{array}\right)E_{x}\left(Y^{k}{\left(1-Y\right)}^{n-k};t_{1},t_{2}\right)\\ \equiv \left(\begin{array}{c} n\\ k \end{array}\right)p_{n,k}\left(t_{1},t_{2}\right) \end{array}$$ (1)

Thus, we must compute the binomial moments of the allele frequency distribution in the ancient population. In the *Appendix*, we show that this can be computed using matrix exponentiation,

$$p_{n,k}\left(t_{1},t_{2}\right)={\left(e^{Qt_{2}}e^{Q^{↓}t_{1}}h_{n}\right)}_{i},$$ (2)

where ${\left(v\right)}_{i}$ indicates the *i*th element of the vector v, $h_{n}={\left({\left(1-x\right)}^{n},x{\left(1-x\right)}^{n-1},\ldots ,x^{n}\right)}^{T}$ and *Q* and $Q^{↓}$ are the sparse matrices

$$Q_{ij}=\{\begin{array}{ll} \frac{1}{2}i\left(i-1\right) & \text{if}\;j=i-1\\ -i\left(n-i\right) & \text{if}\;j=i\\ \frac{1}{2}\left(n-i\right)\left(n-i-1\right) & \text{if}\;j=i+1\\ 0 & \text{else} \end{array}$$

and

$$Q_{ij}^{↓}=\{\begin{array}{ll} \frac{1}{2}i\left(i-1\right) & \text{if}\;j=i-1\\ -i\left(n-i+1\right) & \text{if}\;j=i\\ \frac{1}{2}\left(n-i+1\right)\left(n-i\right) & \text{if}\;j=i+1\\ 0 & \text{else}. \end{array}$$

**Figure 1:**
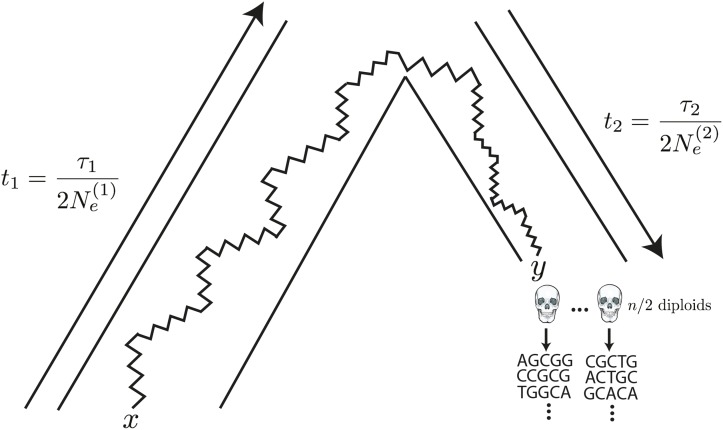
The generative model. Alleles are found at frequency *x* in the modern population, and are at frequency *y* in the ancient population. The modern population has effective size $N_{e}^{\left(1\right)}$ and has evolved for $\tau_{1}$ generations since the common ancestor of the modern and ancient populations, while the ancient population is of size $N_{e}^{\left(2\right)}$ and has evolved for $\tau_{2}$ generations. Ancient diploid samples are taken and sequenced to possibly low coverage, with errors. Arrows indicate that the sampling probability can be calculated by evolving alleles *backward* in time from the modern population, and then forward in time to the ancient population.

This result has an interesting interpretation: the matrix $Q^{↓}$ can be thought of as evolving the allele frequencies back in time, from the modern population to the common ancestor of the ancient and modern populations, while *Q* evolves the allele frequencies forward in time, from the common ancestor to the ancient population (Figure 1).

Because of the fragmentation and degradation of DNA that is inherent in obtaining sequence data from ancient individuals, it is difficult to obtain the high coverage data necessary to make high quality genotype calls from ancient individuals. To address this, we instead work directly with raw read data, and average over all the possible genotypes weighted by their probability of producing the data. Specifically, we follow Nielsen *et al.* (2012) in modeling the probability of the read data in the ancient population, given the allele frequency at site *l* as

$$\mathbb{P}\left(R_{l}|k\right)=\sum_{g_{1,l}=0}^{2}\ldots \sum_{g_{n,l}=0}^{2}I\left(\sum_{i=1}^{m}g_{i,l}=k\right)\prod_{i=1}^{n}\left(\begin{array}{c} 2\\ g_{i,l} \end{array}\right)\mathbb{P}\left(R_{i,l}|g_{i,l}\right),$$

where $R_{i,l}=\left(a_{i,l},d_{i,l}\right)$ are the counts of ancestral and derived reads in individual *i* at site *l*, $g_{i,l}\in \left\{0,1,2\right\}$ indicates the possible genotype of individual *i* at site *l* (*i.e.*, 0 = homozygous ancestral, 1 = heterozygous, 2 = homozygous derived), and $\mathbb{P}\left(R_{i,l}|g_{i,l}\right)$ is the probability of the read data at site *l* for individual *i*, assuming that the individual truly has genotype $g_{i,l}.$ We use a binomial sampling with error model, in which the probability that a truly derived site appears ancestral (and vice versa) is given by ϵ. We emphasize that the parameter ϵ will capture both sequencing error as well as PMD [*cf*. Racimo *et al.* (2016), who found that adding an additional parameter to specifically model PMD does not improve inferences]. Thus,

$$\mathbb{P}\left(R|g\right)=\left(\begin{array}{c} a+d\\ d \end{array}\right)p_{g}^{d}{\left(1-p_{g}\right)}^{a}$$

with

$$p_{0}=\epsilon$$

$$p_{1}=\frac{1}{2}$$

$$p_{2}=1-\epsilon$$

Combining these two aspects together by summing over possible allele frequencies weighted by their probabilities, we obtain our likelihood of the ancient data,

$$L\left(D\right)=\prod_{l=1}^{L}\sum_{k=0}^{n}\mathbb{P}\left(R_{l}|k\right)p_{n,k}\left(x_{l}\right).$$ (3)

### Data availability

The most recent Python implementations of the described methods are available at www.github.com/schraiber/continuity/. A snapshot of the code used as of the publication of the manuscript is available at https://zenodo.org/record/1054127.

## Results

### Impact of coverage and number of samples on inferences

To explore the tradeoff of sequencing more individuals at lower depth compared to fewer individuals at higher coverage, we performed simulations using msprime (Kelleher *et al.* 2016) combined with custom scripts to simulate error and low coverage data. Briefly, we assumed a Poisson distribution of reads at every site with mean given by the coverage, and then simulated reads by drawing from the binomial distribution described in the *Methods*.

**Figure 2:**
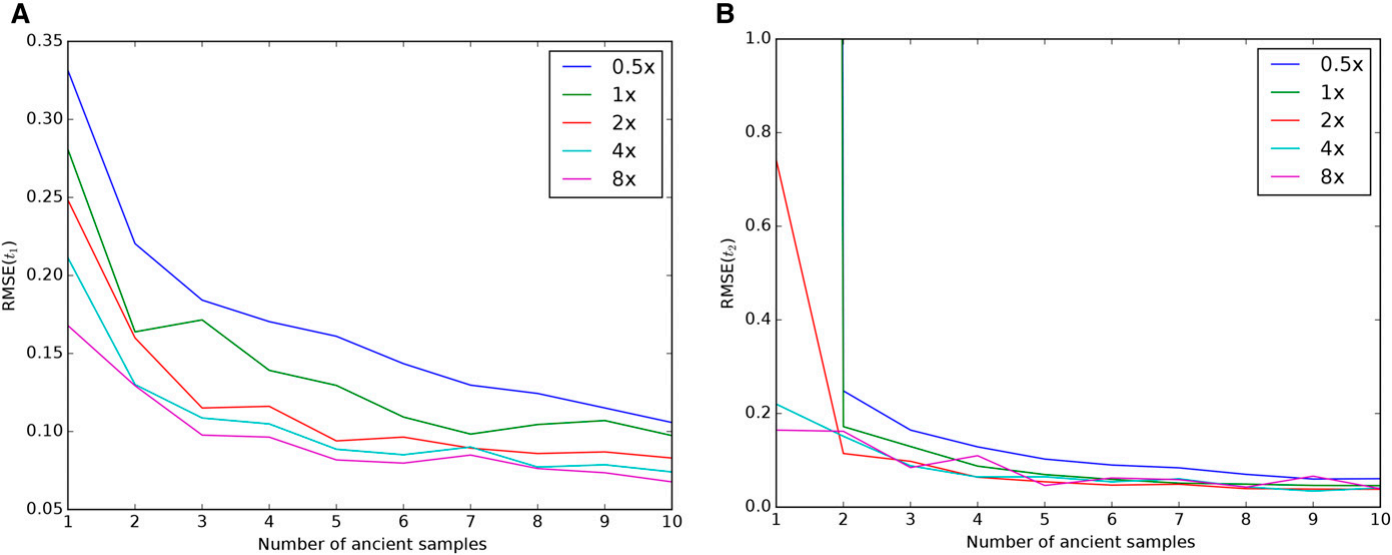
Impact of sampling scheme on parameter estimation error. In each panel, the *x*-axis represents the number of simulated ancient samples, while the *y*-axis shows the relative root mean square error for each parameter. Each different line corresponds to individuals sequenced to different depth of coverage. (A) shows results for $t_{1}$ while (B) shows results for $t_{2}.$ Simulated parameters are $t_{1}=0.02$ and $t_{2}=0.05.$

First, we examined the impact of coverage and number of samples on the ability to recover the drift times in the modern and ancient populations. Figure 2 shows results for data simulated with $t_{1}=0.02$ and $t_{2}=0.05,$ corresponding to an ancient individual who died 300 generations ago from a population of effective size 1000. The populations split 400 generations ago, and the modern population has an effective size of 10,000. We simulated ∼180,000 SNPs by simulating 100,000 500-bp fragments. Inferences of $t_{1}$ can be relatively accurate even with only one low coverage ancient sample (Figure 2A). However, inferences of $t_{2}$ benefit much more from increasing the number of ancient samples, as opposed to coverage (Figure 2B). Supplemental Material, Table S1 shows that there is very little change in the average estimated parameter, indicating that most of the change in RMSE is due to decreased sampling variance. Thus, two individuals sequenced to 0.5× coverage have a much lower error than a single individual sequenced to 2× coverage, even though there is very little bias in either case. To explore this effect further, we derived the sampling probability of alleles covered by exactly one sequencing read (see *Appendix*). We found that sites covered only once have no information about $t_{2},$ suggesting that evidence of heterozygosity is very important for inferences about $t_{2}.$ Finally, though we showed through simulation that there is sufficient power to disentangle $t_{1}$ from $t_{2},$ estimates of these parameters are negatively correlated, due to the necessity of fitting the total drift time $t_{1}+t_{2}$ (Figure S1; all supplementary legends can be found in File S1).

**Figure 3:**
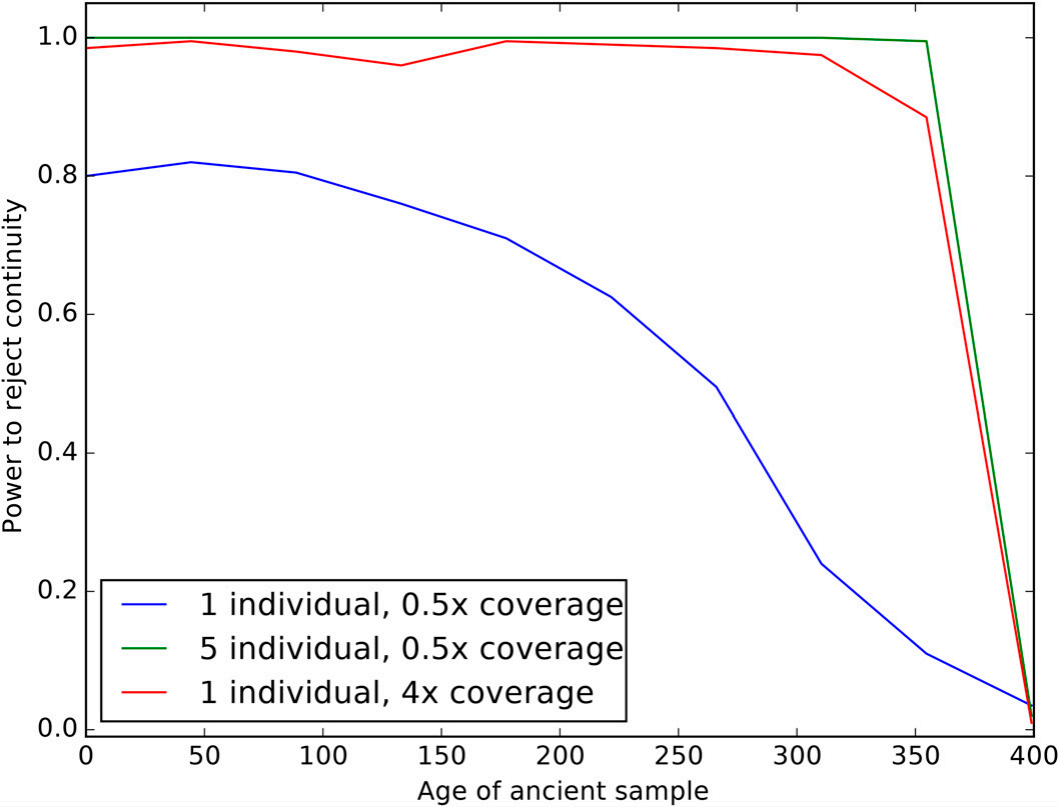
Impact of sampling scheme on rejecting population continuity. The *x*-axis represents the age of the ancient sample in generations, with 0 indicating a modern sample and 400 indicating a sample from exactly at the split time 400 generations ago. The *y*-axis shows the proportion of simulations in which we rejected the null hypothesis of population continuity. Each line shows different sampling schemes, as explained in the legend.

We next examined the impact of coverage and sampling on the power to reject the hypothesis that the ancient individuals came from a population that is directly ancestral to the modern population. We analyzed both low coverage (0.5×) and higher coverage (4×) datasets consisting of one (for both low and high coverage samples) or five individuals (only for low coverage). We simulated data with parameters identical to the previous experiment, except we now examined the impact of varying the age of the ancient sample from 0 generations ago through to the split time with the modern population. We then performed a likelihood ratio test comparing the null model of continuity, in which $t_{2}=0,$ to a model in which the ancient population is not continuous. Figure 3 shows the power of the likelihood ratio test. For a single individual sequenced to low coverage, we see that the test only has power for very recently sampled ancient individuals (*i.e.*, samples that are highly diverged from the modern population). However, the power increases dramatically as the number of individuals or the coverage per individual is increased; sequencing five individuals to 0.5× coverage results in essentially perfect power to reject continuity. Nonetheless, for samples that are very close to the divergence time, it will be difficult to determine if they are ancestral to the modern population or not, because differentiation is incomplete.

### Impact of admixture

**Figure 4:**
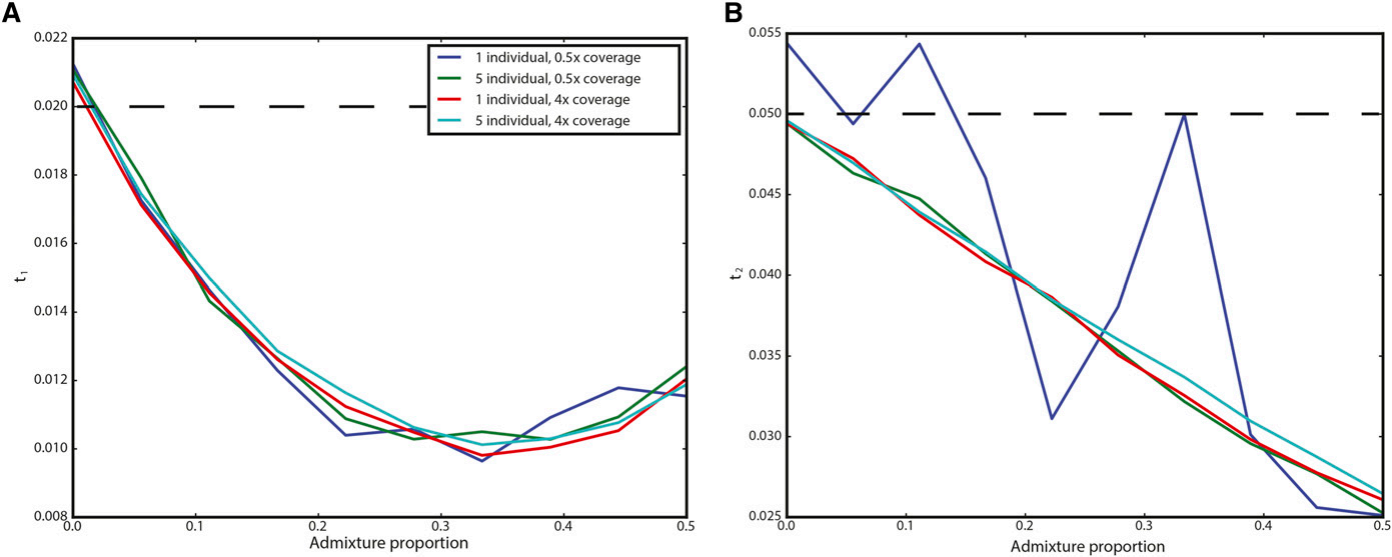
Impact of admixture from the ancient population on inferred parameters. The *x*-axis shows the admixture proportion, and the *y*-axis shows the average parameter estimate across simulations. Each line corresponds to a different sampling strategy, as indicated in the legend. (A) shows results for $t_{1}$ and (B) shows results for $t_{2}.$ The true values of $t_{1}=0.02$ and $t_{2}=0.05$ are indicated by dashed lines.

We examined two possible ways that admixture can result in violations of the model to assess their impact on inference. In many situations, there may have been secondary contact between the population from which the ancient sample is derived and the modern population used as a reference. We performed simulations of this situation by modifying the simulation corresponding to Figure 2 (300-generation-old ancient sample from population of size 1000 split from a population of size 10,000 400 generations ago) to include subsequent admixture from the ancient population to the modern population 200 generations ago (NB: this admixture occurred *more recently* than the ancient sample). In Figure 4, we show the results for admixture proportions ranging from 0 to 50%. Counterintuitively, estimates of $t_{1}$ initially *decrease* before again increasing. This is likely a result of the increased heterozygosity caused by admixture, which acts to artificially inflate the effective size of the modern population, and, thus, decrease $t_{1}.$ As expected, $t_{2}$ is estimated to be smaller the more admixture there is; indeed, for an admixture rate of 100%, the modern and ancient samples are continuous. The impact on $t_{2}$ appears to be linear, and is well approximated by $\left(1-f\right)t_{2}$ if the admixture fraction is *f*.

**Figure 5:**
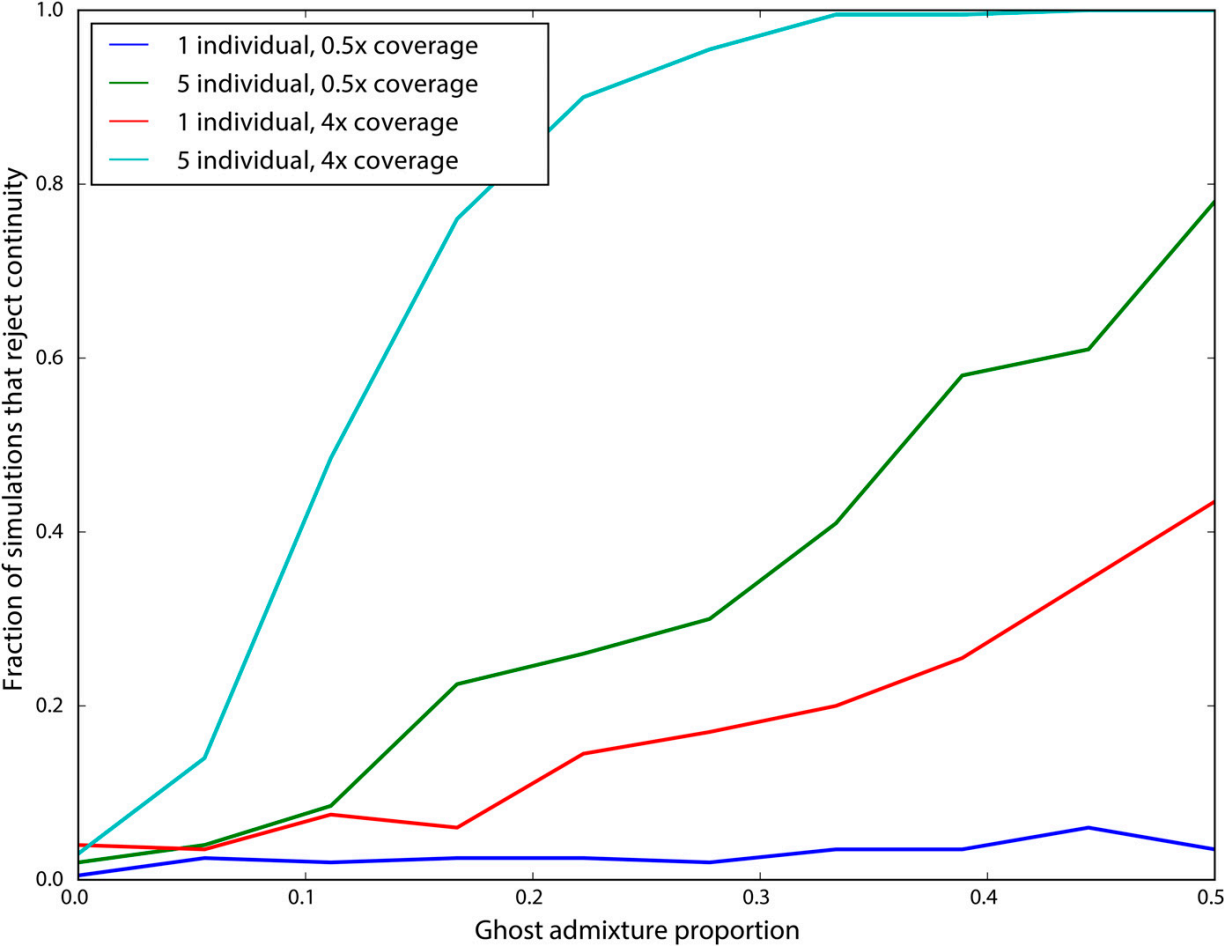
Impact of ghost admixture on rejecting continuity. The *x*-axis shows the admixture proportion from the ghost population, and the *y*-axis shows the fraction of simulations in which continuity was rejected. Each line corresponds to a different sampling strategy, as indicated in the legend.

In other situations, there may be admixture from an unsampled “ghost” population into the modern population. If the ghost admixture is of a high enough proportion, it is likely to cause a sample that is, in fact, a member of a directly ancestral population to appear not to be ancestral. We explored this situation by augmenting our simulations in which the ancient sample is continuous with an outgroup population diverged from the modern population 0.04 time units ago (corresponding to 800 generations ago), and contributed genes to the modern population 0.01 time units ago (corresponding to 200 generations ago). We then assessed the impact on rejecting continuity using the likelihood ratio test (Figure 5). As expected, we see that low-power sampling strategies (such as a single individual sequenced to low coverage) are very minimally impacted by ghost admixture. However, for more powerful sampling strategies, moderate rates of ghost admixture (∼10%) result in rejection of continuity.

### Impact of contamination

**Figure 6:**
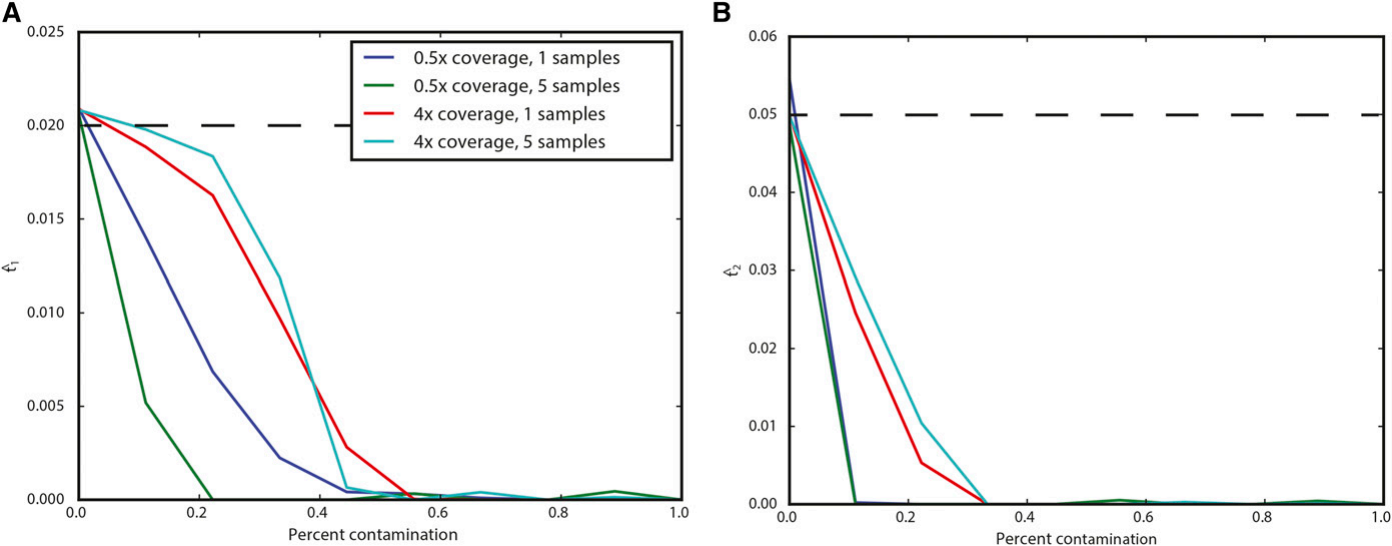
Impact of contamination on parameter inference. The *x*-axis shows the contamination fraction, and the *y*-axis shows the average parameter estimate from simulations. Each line corresponds to a different sampling strategy, as indicated in the legend. (A) shows $t_{1},$ and (B) shows $t_{2}.$ Dashed lines indicate the true values of $t_{1}=0.02$ and $t_{2}=0.05.$

We also explored the impact of foreign DNA contamination on inferences made using this approach. Briefly, we modified the simulations to include a chance *c* of a read being from a modern sample instead of the ancient sample when simulating reads. We again simulated data corresponding to Figure 2, with a 300-generation-old ancient sample from population of size 1000 split from a population of size 10,000 400 generations ago. In Figure 6, we see that relatively modest amounts of contamination can result in estimating zero, or near-zero, drift times. Interestingly, for the same contamination fraction, higher coverage samples are impacted slightly less. Together, this suggests that contamination will result in samples to be falsely inferred to be directly continuous with the modern population.

### Application to ancient humans

We applied our approach to ancient human data from Mathieson *et al.* (2015), which is primarily derived from a SNP capture approach that targeted 1.2 million SNPs. Based on sampling location and associated archeological materials, the individuals were grouped into *a priori* panels, which we used to specify population membership when analyzing individuals together. We analyzed all samples for their relationship to the CEU individuals from the 1000 Genomes Project Consortium (2015). Based on our results, which suggested that extremely low coverage samples would yield unreliable estimates, we excluded panels that are composed of only a single individual sequenced to <2× coverage.

**Figure 7:**
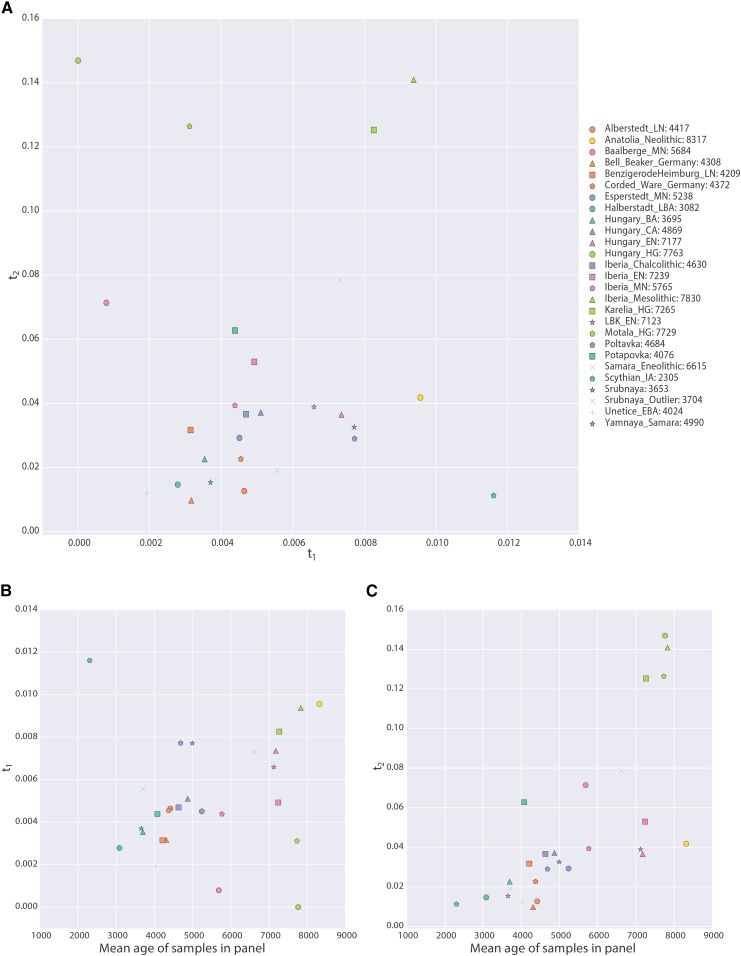
Parameters of the model inferred from ancient West Eurasian samples. (A) shows $t_{1}$ on the *x*-axis and $t_{2}$ on the *y*-axis, with each point corresponding to a population as indicated in the legend. Numbers in the legend correspond to the mean date of all samples in the population. (B and C) Scatterplots of the mean age of the samples in the population (*x*-axis) against $t_{1}$ and $t_{2},$ respectively. Points are described by the same legend as (A).

We computed maximum likelihood estimates of $t_{1}$ and $t_{2}$ for individuals as grouped into populations (Figure 7A and Table 1). We observe that $t_{2}$ is significantly greater than zero for all populations according to the likelihood ratio test. Thus, none of these populations are consistent with directly making up a large proportion of the ancestry of modern CEU individuals. Strikingly, we see that $t_{2}\gg t_{1},$ despite the fact these samples died in the past, and thus they belonged to a lineage that must have existed for fewer generations since the population split than the modern samples. This suggests that all of the ancient populations are characterized by extremely small effective population sizes.

**Table 1:** Details of populations included in analysis

Pop	Cov	Date	$t_{1}$	$t_{2}$	lnL	$t_{1}$ (Cont)	lnL (Cont)
Alberstedt_LN	12.606	4417.000	0.005	0.013	−779,411.494	0.006	−779,440.143
Anatolia_Neolithic	3.551	8317.500	0.010	0.042	−9,096,440.714	0.044	−9,106,156.877
Baalberge_MN	0.244	5684.333	0.001	0.071	−201,575.306	0.007	−201,750.419
Bell_Beaker_Germany	1.161	4308.444	0.003	0.010	−1,834,486.744	0.008	−1,834,652.858
BenzigerodeHeimburg_LN	0.798	4209.750	0.003	0.032	−346,061.545	0.007	−346,134.356
Corded_Ware_Germany	2.250	4372.833	0.005	0.023	−2,139,002.723	0.017	−2,139,858.192
Esperstedt_MN	30.410	5238.000	0.005	0.029	−975,890.329	0.009	−976,047.889
Halberstadt_LBA	5.322	3082.000	0.003	0.015	−558,966.522	0.004	−558,993.078
Hungary_BA	3.401	3695.750	0.004	0.023	−789,754.969	0.010	−789,939.889
Hungary_CA	5.169	4869.500	0.005	0.037	−504,413.094	0.010	−504,549.603
Hungary_EN	4.033	7177.000	0.007	0.036	−3,478,429.262	0.033	−3,481,855.461
Hungary_HG	5.807	7763.000	0.000	0.147	−469,887.471	0.015	−471,652.083
Iberia_Chalcolithic	1.686	4630.625	0.005	0.037	−2,351,769.869	0.028	−2,354,249.543
Iberia_EN	4.875	7239.500	0.005	0.053	−1,483,274.628	0.030	−1,485,675.934
Iberia_MN	5.458	5765.000	0.004	0.039	−1,491,407.962	0.023	−1,492,793.179
Iberia_Mesolithic	21.838	7830.000	0.009	0.141	−720,759.133	0.030	−723,091.935
Karelia_HG	2.953	7265.000	0.008	0.125	−652,952.676	0.033	−655,352.439
LBK_EN	2.894	7123.429	0.007	0.039	−3,656,617.954	0.033	−3,660,838.639
Motala_HG	2.207	7729.500	0.003	0.126	−1,477,338.076	0.068	−1,489,573.895
Poltavka	2.211	4684.500	0.008	0.029	−1,334,662.071	0.020	−1,335,358.630
Potapovka	0.267	4076.500	0.004	0.063	−220,112.816	0.011	−220,251.379
Samara_Eneolithic	0.463	6615.000	0.007	0.078	−362,161.674	0.020	−362,689.209
Scythian_IA	3.217	2305.000	0.012	0.011	−492,961.306	0.013	−492,973.694
Srubnaya	1.662	3653.273	0.004	0.015	−2,578,065.957	0.013	−2,578,645.731
Srubnaya_Outlier	0.542	3704.500	0.006	0.019	−285,828.766	0.008	−285,851.523
Unetice_EBA	1.320	4024.786	0.002	0.012	−1,676,798.610	0.008	−1,677,026.310
Yamnaya_Samara	1.937	4990.500	0.008	0.033	−2,440,183.354	0.028	−2,442,192.801

Pop, population name; cov, mean coverage of individuals in the population; date, mean date of individuals in the population; $t_{1},$ maximum likelihood estimate of $t_{1}$ in the full model; $t_{2},$maximum likelihood estimate of $t_{2}$ in the full model; LnL, maximum likelihood value in the full model; $t_{1}$ (cont), maximum likelihood estimate of $t_{1}$ in the model where $t_{2}=0;$ LnL, maximum likelihood value in the model where $t_{2}=0.$

We further explored the relationship between the dates of the ancient samples and the parameters of the model by plotting $t_{1}$ and $t_{2}$ against the mean sample date of all samples in that population (Figure 7, B and C). We expected to find that $t_{1}$ correlated with sample age, under the assumption that samples were members of relatively short-lived populations that diverged from the “main-stem” of CEU ancestry. Instead, we see no correlation between $t_{1}$ and sample time, suggesting that the relationship of these populations to the CEU is complicated, and not summarized well by the age of the samples. On the other hand, we see a strong positive correlation between $t_{2}$ and sampling time ($P<1\times 10^{-4}$). Because $t_{2}$ is a compound parameter, it is difficult to directly interpret this relationship. However, it is consistent with the most ancient samples belonging to populations with the smallest effective sizes, consistent with previous observations (Skoglund *et al.* 2014).

**Figure 8:**
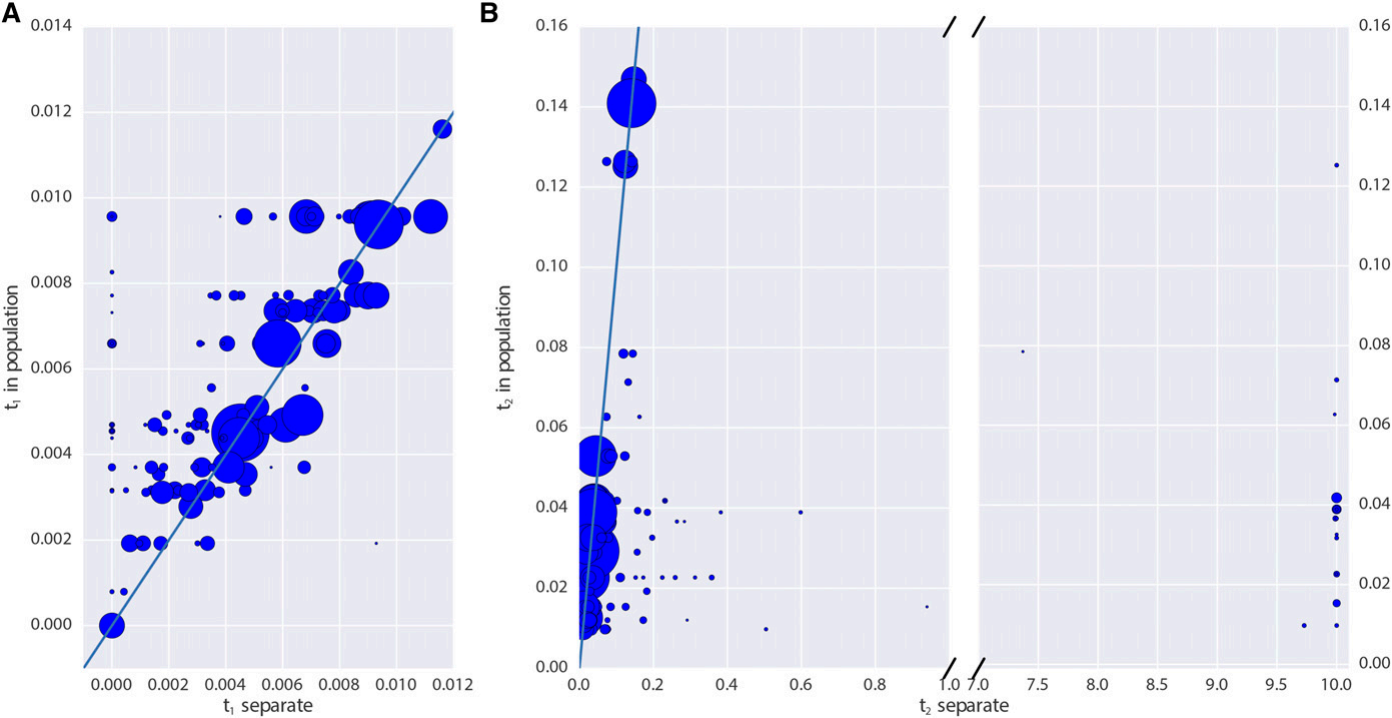
Impact of pooling individuals into populations when estimating model parameters from real data. In both panels, the *x*-axis indicates the parameter estimate when individuals are analyzed separately, while the *y*-axis indicates the parameter estimate when individuals are grouped into populations. Size of points is proportional to the coverage of each individual. (A) reports the impact on estimation of $t_{1},$ while (B) reports the impact on $t_{2}.$ Note that (B) has a broken *x*-axis. Solid lines in each figure indicate y=x.

Finally, we examined the impact of grouping individuals into populations in real data. We see that estimates of $t_{1}$ for low coverage samples are typically lower when analyzed individually than when pooled with other individuals of the same panel (Figure 8A); because Table S1 shows that there is no downward bias in $t_{1}$ for low coverage, this suggests that there may be some heterogeneity in these panels. On the other hand, there is substantial bias toward overestimating $t_{2}$ when analyzing samples individually, particularly for very low coverage samples (Figure 8B). This again shows that, for estimates that rely on heterozygosity in ancient populations, pooling many low coverage individuals can significantly improve estimates.

## Discussion

aDNA presents unique opportunities to enhance our understanding of demography and selection in recent history. However, it also comes equipped with several challenges, due to DNA PMD (Sawyer *et al.* 2012). Several strategies have been developed to deal with the low quality of aDNA data, from relatively simple options like sampling a read at random at every site (Green *et al.* 2010) to more complicated methods making use of genotype likelihoods (Racimo *et al.* 2016). Here, we presented a novel maximum likelihood approach for making inferences about how ancient populations are related to modern populations by analyzing read counts from multiple ancient individuals, and explicitly modeling relationship between the two populations. We explicitly condition on the allele frequency in a modern population; as we show in the *Appendix*, this renders our method robust to ascertainment in modern samples. Thus, it can be used with SNP capture data. Moreover, confidence intervals can be calculated using a nonparametric bootstrap, although this will be computational intensive for large ancient panels, such as those considered in this manuscript. Using this approach, we examined some aspects of sampling strategy for aDNA analysis, and we applied our approach to ancient humans.

We found that sequencing many individuals from an ancient population to low coverage (0.5–1×) can be a significantly more cost-effective strategy than sequencing fewer individuals to relatively high coverage. For instance, we saw from simulations that far more accurate estimates of the drift time in an ancient population can be obtained by pooling two individuals at 0.5× coverage than by sequencing a single individual to 2× coverage (Figure 2). We saw this replicated in our analysis of the real data: low coverage individuals showed a significant amount of variation and bias in estimating the model parameters that was substantially reduced when individuals were analyzed jointly in a population (Figure 8). To explore this further, we showed that sites sequenced to 1× coverage in a single individual retain no information about the drift time in the ancient population. This can be intuitively understood because the drift time in the ancient population is strongly related the amount of heterozygosity in the ancient population: an ancient population with a longer drift time will have lower heterozygosity at sites shared with a modern population. When a site is only sequenced once in a single individual, there is no information about the heterozygosity of that site. We also observed a pronounced upward bias in estimates of the drift time in the ancient population from low coverage samples. We speculate that this is due to the presence of few sites covered more than once being likely to be homozygous, thus deflating the estimate of heterozygosity in the ancient population. Thus, for analysis of SNP data, we recommend that aDNA sampling be conduced to maximize the number of individuals from each ancient population that can be sequenced to ∼1×, rather than attempting to sequence fewer individuals to high coverage. This suggestion can be complicated when samples have vastly different levels of endogenous DNA, where it may be cost effective to sequence high quality samples to higher coverage. In that case, we recommend sequencing samples to at least 3–4× coverage; as evidenced by Figure 2 and Figure 3, single samples at <4× coverage provide extremely limited information about the drift time in the ancient population, and, thus, little power to reject continuity.

When we looked at the impact of model misspecification, we saw several important patterns. First, the influence of admixture from the ancient population on inferences of $t_{2}$ is approximately linear, suggesting that if there are estimates of the amount of admixture between the modern and ancient population, a bias-corrected estimate of $t_{2}$ could be produced (Figure 4B). The impact on inference of $t_{1}$ is more complicated: admixture actually *reduces* estimates of $t_{1}$ (Figure 4A). This is likely because admixture increases the heterozygosity in the modern population, thus causing the amount of drift time to seem reduced. In both cases, the bias is not impacted by details of sampling strategy, although the variance of estimates is highly in a way consistent with Figure 2.

Of particular interest in many studies of ancient populations is the question of direct ancestry: are the ancient samples members of a population that contributed substantially to a modern population? We emphasize that this does not mean that the particular samples were direct ancestors of any modern individuals; indeed, this is exceedingly unlikely for old samples (Donnelly 1983; Chang 1999; Baird *et al.* 2003; Rohde *et al.* 2004). Instead, we are asking whether an ancient sample was a member of a population that is directly continuous with a modern population. Several methods have been proposed to test this question, but thus far they have been limited to many individuals sequenced at a single locus (Sjödin *et al.* 2014) or to a single individual with genome-wide data (Rasmussen *et al.* 2014). Our approach provides a rigorous, maximum-likelihood framework for testing questions of population continuity using multiple low coverage ancient samples. We saw from simulations (Figure 3) that data from single, low coverage individuals result in very little power to reject the null hypothesis of continuity unless the ancient sample is very recent (*i.e.*, it has been diverged from the modern population for a long time). Nonetheless, when low coverage individuals are pooled together, or a single high coverage individual is used, there is substantial power to reject continuity for all but the most ancient samples (*i.e.*, samples dating from very near the population split time).

Because many modern populations may have experienced admixture from unsampled “ghost” populations, we also performed simulations to test the impact of ghost admixture on the probability of falsely rejecting continuity. We find that single ancient samples do not provide sufficient power to reject continuity, even for high levels of ghost admixture, while increasingly powerful sampling schemes, adding more individuals or higher coverage per individual, reject continuity at higher rates. However, in these situations, whether we regard rejection of continuity as a false or true discovery is somewhat subjective: how much admixture from an outside population is required before considering a population to not be directly ancestral? In future work it will be extremely important to estimate the “maximum contribution” of the population an ancient sample comes from (*cf*. Sjödin *et al.* 2014).

To gain new insights from empirical data, we applied our approach to ancient samples throughout Europe. Notably, we rejected continuity for all populations that we analyzed. This is unsurprising, given that European history is extremely complicated, and has been shaped by many periods of admixture (Lazaridis *et al.* 2014, 2016; Haak *et al.* 2015). Thus, modern Europeans have experienced many periods of “ghost” admixture (relative to any particular ancient sample). Nonetheless, our results show that none of these populations are even particularly close to directly ancestral, as our simulations have shown that rejection of continuity will not occur with low levels of ghost admixture.

Second, we observed that the drift time in the ancient population was much larger than the drift time in the modern population. Assuming that the ancient sample were a contemporary sample, the ratio $t_{1}/t_{2}$ is an estimator of the ratio $N_{e}^{\left(2\right)}/N_{e}^{\left(1\right)};$ in fact, because the ancient sample existed for fewer generations since the common ancestor of the ancient and modern populations, $t_{1}/t_{2}$ acts as an upper bound on $N_{e}^{\left(2\right)}/N_{e}^{\left(1\right)}.$ Moreover, this is unlikely to be due to unmodeled error in the ancient samples: error would be expected increase the heterozygosity in the ancient sample, and thus *decrease* our estimates of $t_{2}.$ Another potential complication is the fact that modern Europeans are a mixture of multiple ancestral populations (Lazaridis *et al.* 2014; Haak *et al.* 2015). As shown through simulation, admixture increases heterozygosity in the modern population and thus decreases estimates of $t_{1}.$ However, even very large amounts of ghost admixture did not result in the order-of-magnitude differences we see in the real data, suggesting that ghost admixture cannot account for all the discrepancy between modern and ancient $N_{e}.$ Thus, we find strong support for the observation that ancient Europeans were often members of small, isolated populations (Skoglund *et al.* 2014). We interpret these two results together as suggestive that many ancient samples found thus far in Europe were members of small populations that ultimately went locally extinct. Nonetheless, there may be many samples that belonged to larger metapopulations, and further work is necessary to specifically examine those cases.

We further examined the effective sizes of ancient populations through time by looking for a correlation between the age of the ancient populations and the drift time leading to them (Figure 7C). We saw a strong positive correlation, and, although this drift time is a compound parameter, which complicates interpretations, it appears that the oldest Europeans were members of the smallest populations, and that effective population size has grown through time as agriculture spread through Europe.

We anticipate the further development of methods that explicitly account for differential drift times in ancient and modern samples will become important as aDNA research becomes even more integrated into population genomics. This is because many common summary methods, such as the use of Structure (Pritchard *et al.* 2000) and Admixture (Alexander *et al.* 2009), are sensitive to differential amounts of drift between populations (Falush *et al.* 2016). As we have shown in ancient Europeans, ancient samples tend to come from isolated subpopulations with a large amount of drift, thus confounding such summary approaches. Moreover, standard population genetics theory shows that allele frequencies are expected to be deterministically lower in ancient samples, even if they are direct ancestors of a modern population. Intuitively, this arises because the alleles must have arisen at some point from new mutations, and thus were at lower frequencies in the past. A potentially fruitful avenue to combine these approaches moving forward may be to separate regions of the genome based on ancestry components, and assess the ancestry of ancient samples relative to specific ancestry components, rather than to genomes as a whole.

Our current approach leaves several avenues for improvement. We use a relatively simple error model that wraps up both PMD and sequencing error into a single parameter. While Racimo *et al.* (2016) shows that adding an additional parameter for PMD-related error does not significantly change results, the recent work of Kousathanas *et al.* (2017) shows that building robust error models is challenging and essential to estimating heterozygosity properly. Although our method is robust to nonconstant demography because we consider only alleles that are segregating in both the modern and the ancient population, we are losing information by not modeling new mutations that arise in the ancient population. Similarly, we only consider a single ancient population at a time, albeit with multiple samples. Ideally, ancient samples would be embedded in complex demographic models that include admixture, detailing their relationships to each other and to modern populations (Patterson *et al.* 2012; Lipson and Reich 2017). However, inference of such complex models is difficult, and, though there has been some progress in simplified cases (Pickrell and Pritchard 2012; Lipson *et al.* 2014), it remains an open problem due to the difficult of simultaneously inferring a nontree-like topology along with demographic parameters. Software such as momi (Kamm *et al.* 2017), which can compute the likelihood of SNP data in an admixture graph, may be able to be used to integrate over genotype uncertainty in larger settings than considered here.

## Supplementary Material

Supplemental material is available online at www.genetics.org/lookup/suppl/doi:10.1534/genetics.117.300448/-/DC1.

## Appendix

### Computing Allele Frequency Moments in the Ancient Population

We wish to compute moments of the form

$$E_{x}\left(g\left(Y\right);t_{1},t_{2}\right)=\int_{0}^{1}g\left(y\right)f\left(y;x,t_{1},t_{2}\right)dy$$ (4)

To do so, we make use of several results from diffusion theory. To ensure that this paper is self-contained, we briefly review those results here. The interested reader may find much of this material covered in Karlin and Taylor (1981) and Ewens (2012). Several similar calculations can be found in Griffiths (2003).

Let the probability of an allele going from frequency *x* to frequency *y* in *τ* generations in a population of size $N_{e}$ be f(x,y;t), where $t=\tau /\left(2N_{e}\right).$ Under a wide variety of models, the change in allele frequencies through time is well approximated by the Wright-Fisher diffusion, which is characterized by its generator,

$$ℒ=\frac{1}{2}x\left(1-x\right)\frac{d^{2}}{dx^{2}}.$$

The generator of a diffusion process is useful, because it can be used to define a differential equation for the moments of that process,

$$\frac{d}{dt}E_{x}\left(g\left(X_{t}\right)\right)=E_{x}\left(ℒg\left(X_{t}\right)\right).$$ (5)

We will require the *speed measure* of the Wright-Fisher diffusion, $m\left(x\right)=x^{-1}{\left(1-x\right)}^{-1},$ which essentially describes how slow a diffusion at position *x* is “moving” compared to a Brownian motion at position *x*. Note that all diffusions are reversible with respect to their speed measures, *i.e.*

m(x)f(x,y;t)=m(y)f(y,x;t).

We additionally require the probability of loss, *i.e.* the probability that the allele currently at frequency *x* is ultimately lost from the population. This is

$$u_{0}\left(x\right)=1-x.$$

Note that it is possible to condition the Wright-Fisher diffusion to eventually be lost. The transition density can be computed as

$$f^{↓}\left(x,y;t\right)=f\left(x,y;t\right)\frac{u_{0}\left(y\right)}{u_{0}\left(x\right)}$$

by using Bayes theorem. The diffusion conditioned on loss is characterized by its generator,

$${ℒ}^{↓}=-x\frac{d}{dx}+\frac{1}{2}x\left(1-x\right)\frac{d^{2}}{dx^{2}}.$$

In an infinite sites model, in which mutations occur at the times of a Poisson process with rate θ/2, and then each drift according to the Wright-Fisher diffusion, a quasi-equilibrium distribution will be reached, known as the frequency spectrum. The frequency spectrum, φ(x), predicts the number of sites at frequency *x*, and can be written in terms of the speed measure and the probability of loss,

$$\varphi \left(x\right)=\theta m\left(x\right)u_{0}\left(x\right).$$

To proceed with calculating (4), note that the conditional probability of an allele being at frequency *y* in the ancient population, given that it is at frequency *x* in the modern population, can be calculated as

$$f\left(y;x,t_{1},t_{2}\right)=\frac{f\left(x,y;t_{1},t_{2}\right)}{\varphi \left(x\right)}$$

where $f\left(x,y;t_{1},t_{2}\right)$ is the joint probability of the allele frequencies in the modern and ancient populations, and φ(x) is the frequency spectrum in the modern population.

Assuming that the ancestral population of the modern and ancient samples was at equilibrium, the joint distribution of allele frequencies can be computed by sampling alleles from the frequency spectrum of the ancestor, and evolving them forward in time via the Wright-Fisher diffusion. This can be written mathematically as

$$f\left(x,y;t_{1},t_{2}\right)=\int_{0}^{1}f\left(z,x;t_{1}\right)f\left(z,y;t_{2}\right)\varphi \left(z\right)dz.$$

We now expand the frequency spectrum in terms of the speed measure and the probability of loss and use reversibility with respect to the speed measure to rewrite the equation,

$$\begin{array}{c} \int_{0}^{1}f\left(z,x;t_{1}\right)f\left(z,y;t_{2}\right)\varphi \left(z\right)dz=\theta \int_{0}^{1}f\left(z,x;t_{1}\right)f\left(z,y;t_{2}\right)m\left(z\right)u_{0}\left(z\right)dz\\ =\theta \int_{0}^{1}\frac{m\left(x\right)}{m\left(z\right)}f\left(x,z;t_{1}\right)f\left(z,y;t_{2}\right)m\left(z\right)u_{0}\left(z\right)dz\\ =\theta m\left(x\right)u_{0}\left(x\right)\int_{0}^{1}f\left(x,z;t_{1}\right)\frac{u_{0}\left(z\right)}{u_{0}\left(x\right)}f\left(z,y;t_{2}\right)dz\\ =\varphi \left(x\right)\int_{0}^{1}f^{↓}\left(x,z;t_{1}\right)f\left(z,y;t_{2}\right)dz. \end{array}$$

The third line follows by multiplying by $u_{0}\left(x\right)/u_{0}\left(x\right)=1.$ This equation has the interpretation of sampling an allele from the frequency spectrum in the modern population, then evolving it *backward* in time to the common ancestor, before evolving it *forward* in time to the ancient population. The interpretation of the diffusion conditioned on loss as evolving backward in time arises by considering the fact that alleles arose from unique mutations at some point in the past; hence, looking backward, alleles must eventually be lost at some point in the past.

To compute the expectation, we substitute this form for the joint probability into (4),

$$\begin{array}{c} \int_{0}^{1}g\left(y\right)f\left(y;x,t_{1},t_{2}\right)dy=\int_{0}^{1}g\left(y\right)\left(\int_{0}^{1}f^{↓}\left(x,z;t_{1}\right)f\left(z,y;t_{2}\right)dz\right)dy\\ =\int_{0}^{1}\left(\int_{0}^{1}g\left(y\right)f\left(z,y;t_{2}\right)dy\right)f^{↓}\left(x,z;t_{1}\right)dz, \end{array}$$

where the second line follows by rearranging terms and exchanging the order of integration. Note that this formula takes the form of nested expectations. Specifically,

$$\begin{array}{c} \int_{0}^{1}g\left(y\right)f\left(z,y;t_{2}\right)dy=E_{z}\left(g\left(Y_{t_{2}}\right)\right)\\ \equiv h\left(z\right) \end{array}$$

and

$$\begin{array}{c} \int_{0}^{1}h\left(z\right)f^{↓}\left(x,z;t_{1}\right)dz=E_{x}^{↓}\left(h\left(Z_{t_{1}}\right)\right)\\ =E_{x}\left(g\left(Y\right);t_{1},t_{2}\right). \end{array}$$

We now use (5) to note that

$$\frac{d}{dt}p_{n,k}=\frac{k\left(k-1\right)}{2}p_{n,k-1}-k\left(n-k\right)p_{n,k}+\frac{\left(n-k\right)\left(n-k-1\right)}{2}p_{n,k+1}$$

and

$$\frac{d}{dt}p_{n,k}^{↓}=\frac{k\left(k-1\right)}{2}p_{n,k-1}^{↓}-k\left(n-k+1\right)p_{n,k}^{↓}+\frac{\left(n-k+1\right)\left(n-k\right)}{2}p_{n,k+1}^{↓}$$

with obvious boundary conditions $p_{n,k}\left(0;z\right)=z^{k}{\left(1-z\right)}^{n-k}$ and $p_{n,k}^{↓}\left(0;x\right)=x^{k}{\left(1-x\right)}^{n-k}.$

These systems of differential equations can be rewritten as matrix differential equations with coefficient matrices *Q* and $Q^{↓}$ respectively. Because they are linear, first order equations, they can be solved by matrix exponentiation. Because the expectation of a polynomial in the Wright-Fisher diffusion remains a polynomial, the nested expectations can be computed via matrix multiplication of the solutions to these differential equations, yielding the formula (2).

### Robustness to Ascertainment in the Modern Population

By conditioning on the allele frequency in the modern population, we gain the power to make inferences that are robust to ascertainment in the modern population. To see this, note from Equation 3 in Nielsen and Signorovitch (2003) that

$$f\left(x|A\right)=\frac{f\left(x,A\right)}{f\left(A\right)}$$

where *A* indicates the event that the allele was ascertained in the modern population. A simple generalization of this shows that

$$f\left(x,y|A\right)=\frac{f\left(x,y,A\right)}{f\left(A\right)}.$$

So,

$$\begin{array}{c} f\left(y|x,A\right)=\frac{f\left(x,y|A\right)}{f\left(x|A\right)}\\ =\frac{f\left(x,y,A\right)}{f\left(x,A\right)}\\ =\frac{f\left(A|x,y\right)f\left(x,y\right)}{f\left(A|x\right)f\left(x\right)}\\ =\frac{f\left(x,y\right)}{f\left(x\right)} \end{array}$$

where the final line follows by recognizing that f(A|x,y)=f(A|x) since the allele was ascertained in the modern population. Thus, we see that the ascertainment is removed by conditioning, and we recover the original formula. Note that the robustness to ascertainment is only exact if the allele is ascertained in the modern population, but is expected to be very close to true, so long as the allele is ascertained in a population closer to the modern population than to the ancient population.

### Sites Covered Exactly Once Have no Information About Drift in the Ancient Population

Consider a simplified model in which each site has exactly one read. When we have sequence from only a single individual, we have a set $l_{a}$ of sites where the single read is an ancestral allele, and a set $l_{d}$ of sites where the single read is a derived allele. Thus, we can rewrite (3) as

$$L\left(D\right)=\prod_{l\in l_{a}}\left((1-\epsilon P_{2,0}x_{l})+\frac{1}{2}P_{2,1}\left(x_{l}\right)+\epsilon P_{2,2}\left(x_{l}\right)\right)\prod_{l\in l_{d}}\left(\epsilon P_{2,0}\left(x_{l}\right)+\frac{1}{2}P_{2,1}\left(x_{l}\right)+\left(1-\epsilon \right)P_{2,2}\left(x_{l}\right)\right).$$

We can use formulas from Racimo *et al.* (2016) to compute $P_{2,k}\left(x_{l}\right)$ for k∈{0,1,2},

$$P_{2,0}\left(x_{l}\right)=1-x_{l}e^{-t_{1}}-\frac{1}{2}x_{l}e^{-\left(t_{1}+t_{2}\right)}+x_{l}\left(x_{l}-\frac{1}{2}\right)e^{-\left(3t_{1}+t_{2}\right)}$$

$$P_{2,1}\left(x_{l}\right)=x_{l}e^{-\left(t_{1}+t_{2}\right)}+x_{l}\left(1-2x_{l}\right)e^{-\left(3t_{1}+t_{2}\right)}$$

$$P_{2,2}\left(x_{l}\right)=x_{l}e^{-t_{1}}-\frac{1}{2}x_{l}e^{-\left(t_{1}+t_{2}\right)}+x_{l}\left(x_{l}-\frac{1}{2}\right)e^{-\left(3t_{1}-t_{2}\right)}.$$

Note then that

$$\left(1-\epsilon \right)P_{2,0}\left(x_{l}\right)+\frac{1}{2}P_{2,1}\left(x_{l}\right)+\epsilon P_{2,2}\left(x_{l}\right)=1-\epsilon -x\left(1-2\epsilon \right)e^{-t_{1}}$$

and

$$\epsilon P_{2,0}\left(x_{l}\right)+\frac{1}{2}P_{2,1}\left(x_{l}\right)+\left(1-\epsilon \right)P_{2,2}\left(x_{l}\right)=\epsilon +x\left(1-2\epsilon \right)e^{-t_{1}}.$$

Neither of these formulas depend on $t_{2};$ hence, there is no information about the drift time in the ancient population from data that is exactly 1× coverage.

## References

[bib1] Alexander D. H. Novembre J. Lange K., 2009 Fast model-based estimation of ancestry in unrelated individuals. Genome Res. 19: 1655–1664. 19648217 10.1101/gr.094052.109 PMC2752134

[bib2] Allentoft M. E. Sikora M. Sjögren K. G. Rasmussen S. Rasmussen M., 2015 Population genomics of bronze age Eurasia. Nature 522: 167–172. 26062507 10.1038/nature14507

[bib3] Baird S. J. E. Barton N. H. Etheridge A. M., 2003 The distribution of surviving blocks of an ancestral genome. Theor. Popul. Biol. 64: 451–471. 14630482 10.1016/s0040-5809(03)00098-4

[bib4] Chang J. T., 1999 Recent common ancestors of all present-day individuals. Adv. Appl. Probab. 31: 1002–1026.

[bib5] Dabney J. Meyer M. Pääbo S., 2013 Ancient DNA damage. Cold Spring Harb. Perspect. Biol. 5: a012567. 23729639 10.1101/cshperspect.a012567 PMC3685887

[bib6] Donnelly K. P., 1983 The probability that related individuals share some section of genome identical by descent. Theor. Popul. Biol. 23: 34–63. 6857549 10.1016/0040-5809(83)90004-7

[bib7] Ewens W. J., 2012 Mathematical Population Genetics 1: Theoretical Introduction, Vol. 27. Springer Science & Business Media, Berlin.

[bib8] Falush D. van Dorp L. Lawson D., 2016 A tutorial on how (not) to over-interpret structure/admixture bar plots. bioRxiv https://doi.org/10.1101/066431. 10.1038/s41467-018-05257-7 PMC6092366 30108219

[bib9] Fu Q. Posth C. Hajdinjak M. Petr M. Mallick S., 2016 The genetic history of ice age Europe. Nature 534: 200–205. 27135931 10.1038/nature17993 PMC4943878

[bib10] 1000 Genomes Project Consortium, 2015 A global reference for human genetic variation. Nature 526: 68–74. 26432245 10.1038/nature15393 PMC4750478

[bib11] Green R. E. Krause J. Briggs A. W. Maricic T. Stenzel U., 2010 A draft sequence of the Neandertal genome. Science 328: 710–722. 20448178 10.1126/science.1188021 PMC5100745

[bib12] Griffiths R. C., 2003 The frequency spectrum of a mutation, and its age, in a general diffusion model. Theor. Popul. Biol. 64: 241–251. 12948685 10.1016/s0040-5809(03)00075-3

[bib13] Haak W. Lazaridis I. Patterson N. Rohland N. Mallick S., 2015 Massive migration from the steppe was a source for Indo-European languages in Europe. Nature 522: 207–211. 25731166 10.1038/nature14317 PMC5048219

[bib14] Jewett E. M. Steinrücken M. Song Y. S., 2016 The effects of population size histories on estimates of selection coefficients from time-series genetic data. Mol. Biol. Evol. 33: 3002–3027. 27550904 10.1093/molbev/msw173 PMC5062326

[bib15] Jónsson H. Ginolhac A. Schubert M. Johnson P. L. Orlando L., 2013 mapdamage2.0: fast approximate Bayesian estimates of ancient DNA damage parameters. Bioinformatics 29: 1682–1684. 23613487 10.1093/bioinformatics/btt193 PMC3694634

[bib16] Kamm J. A. Terhorst J. Song Y. S., 2017 Efficient computation of the joint sample frequency spectra for multiple populations. J. Comput. Graph. Stat. 26:182–194. 28239248 10.1080/10618600.2016.1159212 PMC5319604

[bib17] Karlin S. Taylor H. E., 1981 A Second Course in Stochastic Processes. Elsevier, Amsterdam.

[bib18] Kelleher J. Etheridge A. M. McVean G., 2016 Efficient coalescent simulation and genealogical analysis for large sample sizes. PLOS Comput. Biol. 12: e1004842. 27145223 10.1371/journal.pcbi.1004842 PMC4856371

[bib19] Korneliussen T. S. Albrechtsen A. Nielsen R., 2014 ANGSD: analysis of next generation sequencing data. BMC Bioinformatics 15: 356. 25420514 10.1186/s12859-014-0356-4 PMC4248462

[bib20] Kousathanas A. Leuenberger C. Link V. Sell C. Burger J., 2017 Inferring heterozygosity from ancient and low coverage genomes. Genetics 205: 317–332. 27821432 10.1534/genetics.116.189985 PMC5223511

[bib21] Lazaridis I. Patterson N. Mittnik A. Renaud G. Mallick S., 2014 Ancient human genomes suggest three ancestral populations for present-day Europeans. Nature 513: 409–413. 25230663 10.1038/nature13673 PMC4170574

[bib22] Lazaridis I. Nadel D. Rollefson G. Merrett D C. Rohland N., 2016 Genomic insights into the origin of farming in the ancient near east. Nature 536: 419–424. 27459054 10.1038/nature19310 PMC5003663

[bib23] Lipson M. Reich D., 2017 A working model of the deep relationships of diverse modern human genetic lineages outside of Africa. Mol. Biol. Evol. 34: 889–902. 28074030 10.1093/molbev/msw293 PMC5400393

[bib24] Lipson M. Loh P.-R. Patterson N. Moorjani P. Ko Y.-C., 2014 Reconstructing Austronesian population history in island Southeast Asia. Nat. Commun. 5: 4689. 25137359 10.1038/ncomms5689 PMC4143916

[bib25] Mathieson I. Lazaridis I. Rohland N. Mallick S. Patterson N., 2015 Genome-wide patterns of selection in 230 ancient Eurasians. Nature 528: 499–503. 26595274 10.1038/nature16152 PMC4918750

[bib26] Meyer M. Kircher M. Gansauge M. T. Li H. Racimo F., 2012 A high-coverage genome sequence from an archaic Denisovan individual. Science 338: 222–226. 22936568 10.1126/science.1224344 PMC3617501

[bib27] Nielsen R. Signorovitch J., 2003 Correcting for ascertainment biases when analyzing SNP data: applications to the estimation of linkage disequilibrium. Theor. Popul. Biol. 63: 245–255. 12689795 10.1016/s0040-5809(03)00005-4

[bib28] Nielsen R. Korneliussen T. Albrechtsen A. Li Y. Wang J., 2012 SNP calling, genotype calling, and sample allele frequency estimation from new-generation sequencing data. PLoS One 7: e37558. 22911679 10.1371/journal.pone.0037558 PMC3404070

[bib29] Patterson N. Moorjani P. Luo Y. Mallick S. Rohland N., 2012 Ancient admixture in human history. Genetics 192: 1065–1093. 22960212 10.1534/genetics.112.145037 PMC3522152

[bib30] Peter B. M., 2016 Admixture, population structure, and F-statistics. Genetics 202: 1485–1501. 26857625 10.1534/genetics.115.183913 PMC4905545

[bib31] Pickrell J. K. Pritchard J. K., 2012 Inference of population splits and mixtures from genome-wide allele frequency data. PLoS Genet. 8: e1002967. 23166502 10.1371/journal.pgen.1002967 PMC3499260

[bib32] Pinhasi R. Fernandes D. Sirak K. Novak M. Connell S., 2015 Optimal ancient DNA yields from the inner ear part of the human petrous bone. PLoS One 10: e0129102. 26086078 10.1371/journal.pone.0129102 PMC4472748

[bib33] Pritchard J. K. Stephens M. Donnelly P., 2000 Inference of population structure using multilocus genotype data. Genetics 155: 945–959. 10835412 10.1093/genetics/155.2.945 PMC1461096

[bib34] Racimo F. Renaud G. Slatkin M., 2016 Joint estimation of contamination, error and demography for nuclear DNA from ancient humans. PLoS Genet. 12: e1005972. 27049965 10.1371/journal.pgen.1005972 PMC4822957

[bib35] Rasmussen M. Anzick S. L. Waters M. R. Skoglund P. DeGiorgio M., 2014 The genome of a late Pleistocene human from a clovis burial site in western Montana. Nature 506: 225–229. 24522598 10.1038/nature13025 PMC4878442

[bib36] Rohde D. L. Olson S. Chang J. T. 2004 Modelling the recent common ancestry of all living humans. Nature 431: 562–566. 15457259 10.1038/nature02842

[bib37] Sawyer S. Krause J. Guschanski K. Savolainen V. Pääbo S., 2012 Temporal patterns of nucleotide misincorporations and DNA fragmentation in ancient DNA. PLoS One 7: e34131. 22479540 10.1371/journal.pone.0034131 PMC3316601

[bib38] Schraiber J. G. Evans S. N. Slatkin M. 2016 Bayesian inference of natural selection from allele frequency time series. Genetics 203: 493–511. 27010022 10.1534/genetics.116.187278 PMC4858794

[bib39] Sjödin P. Skoglund P. Jakobsson M., 2014 Assessing the maximum contribution from ancient populations. Mol. Biol. Evol. 31: 1248–1260. 24497031 10.1093/molbev/msu059

[bib40] Skoglund P. Malmström H. Raghavan M. Storå J. Hall P., 2012 Origins and genetic legacy of Neolithic farmers and hunter-gatherers in Europe. Science 336: 466–469. 22539720 10.1126/science.1216304

[bib41] Skoglund P. Malmström H. Omrak A. Raghavan M. Valdiosera C., 2014 Genomic diversity and admixture differs for stone-age Scandinavian foragers and farmers. Science 344: 747–750. 24762536 10.1126/science.1253448

