# Supplementary figures and images for: Assessing the Relationship of Ancient and Modern Populations
Source: Genetics. 2017 Nov 22;208(1):383–98. doi: 10.1534/genetics.117.300448 (PMC5753871; doi:10.1534/genetics.117.300448)

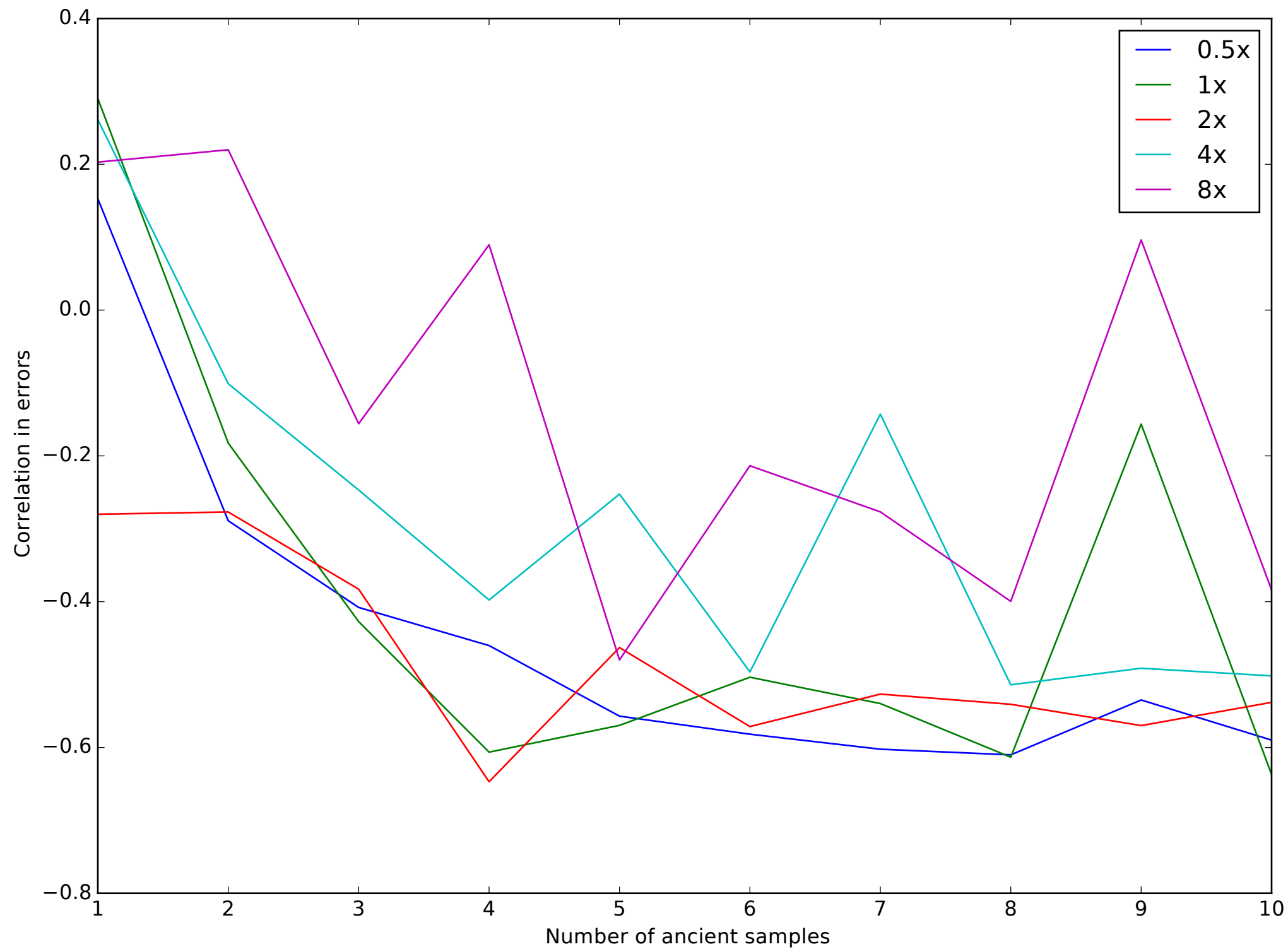

Supplement: Supplementary file 1 [file 383FigureS1.pdf]
