# Supplementary material for: Assessing the Relationship of Ancient and Modern Populations
Source: Genetics. 2017 Nov 22;208(1):383–98. doi: 10.1534/genetics.117.300448 (PMC5753871; doi:10.1534/genetics.117.300448)
Supplement: Supplementary file 3 [file 383FileS1.pdf]

Supplementary Figure 1: Correlation of the error in estimating t_1 and t_2. The x-axis shows the number of ancient individuals sampled, and the y-axis shows the correlation in errors across n simulations, i.e. $\frac{1}{n-1}\sum_{i=1}^{n}(\hat{t_1}_i - t_1)(\hat{t_2}_i - t_2)$. Different lines correspond to different sampling strategies, as indicated in the legend.

Supplementary Table 1: Break down of mean and variance of estimates of t_1 and t_2 under different sampling strategies. "t1mean" and "t2mean" indicate the average value of the MLE across simulations; "t1sd" and "t2sd" indicate the standard deviation of the MLE across simulations.
